# Role of *YES1* amplification in *EGFR* mutation‐positive non‐small cell lung cancer: Primary resistance to afatinib in a patient

**DOI:** 10.1111/1759-7714.13583

**Published:** 2020-08-03

**Authors:** Junyan Tao, Dantong Sun, Helei Hou

**Affiliations:** ^1^ Precision Medicine Center of Oncology The Affiliated Hospital of Qingdao University Qingdao China

**Keywords:** Afatinib, *EGFR*, NSCLC, primary resistance, *YES1*

## Abstract

Epidermal growth factor receptor (EGFR) mutation‐positive non‐small cell lung cancer (NSCLC) patients benefit from EGFR tyrosine kinase inhibitors (TKIs), while some patients demonstrate a resistance to EGFR‐TKIs. In the case reported here, the NSCLC patient harboring an *EGFR*‐sensitive mutation and *YES1* amplification was treated with afatinib as first‐line therapy, but was found to have progressive disease four weeks later. During subsequent chemotherapy, this patient's disease progressed rapidly. Mechanisms of primary resistance to EGFR‐TKIs remain unclear. This case suggested that *YES1* amplification might be associated with primary resistance to EGFR‐TKIs and *YES1* amplification might be a negative predictor of EGFR‐TKI treatment in NSCLC patients harboring *EGFR* sensitive mutations.

## Graphical abstract

This case suggested that *YES1* amplification might be associated with primary resistance to EGFR‐TKIs. *YES1* amplification might be a negative predictor of EGFR‐TKIs treatment in NSCLC patients harboring EGFR sensitive mutation.

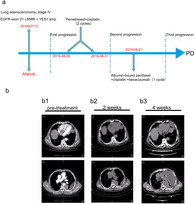

## Introduction

Lung cancer is the leading cause of cancer mortality worldwide. Non‐small cell lung cancer (NSCLC) comprises up to 90% of all lung cancers. Conventional treatment for advanced NSCLC consists of chemotherapy which only has a small impact on patient survival. Molecular targets, such as epidermal growth factor receptor (EGFR), which is involved in cell signaling have led to the development of new, targeted therapies. Patients with EGFR mutation‐positive NSCLC have been reported to benefit from EGFR tyrosine kinase inhibitors (TKIs),^1,2^ although some patients demonstrate a resistance to this treatment. In the case reported here, the patient was diagnosed with NSCLC harboring an *EGFR*‐sensitive mutation and *YES1* amplification and was treated with afatinib as first‐line therapy. However, he was determined to have progressive disease four weeks later and even after subsequent chemotherapy, his disease progressed rapidly. Mechanisms of primary resistance to EGFR‐TKIs remain unclear. This case suggested that *YES1* amplification might be associated with primary resistance to EGFR‐TKIs and that *YES1* amplification might be a negative predictor of EGFR‐TKI treatment in NSCLC patients harboring *EGFR* sensitive mutations.

## Case report

**Figure 1:**
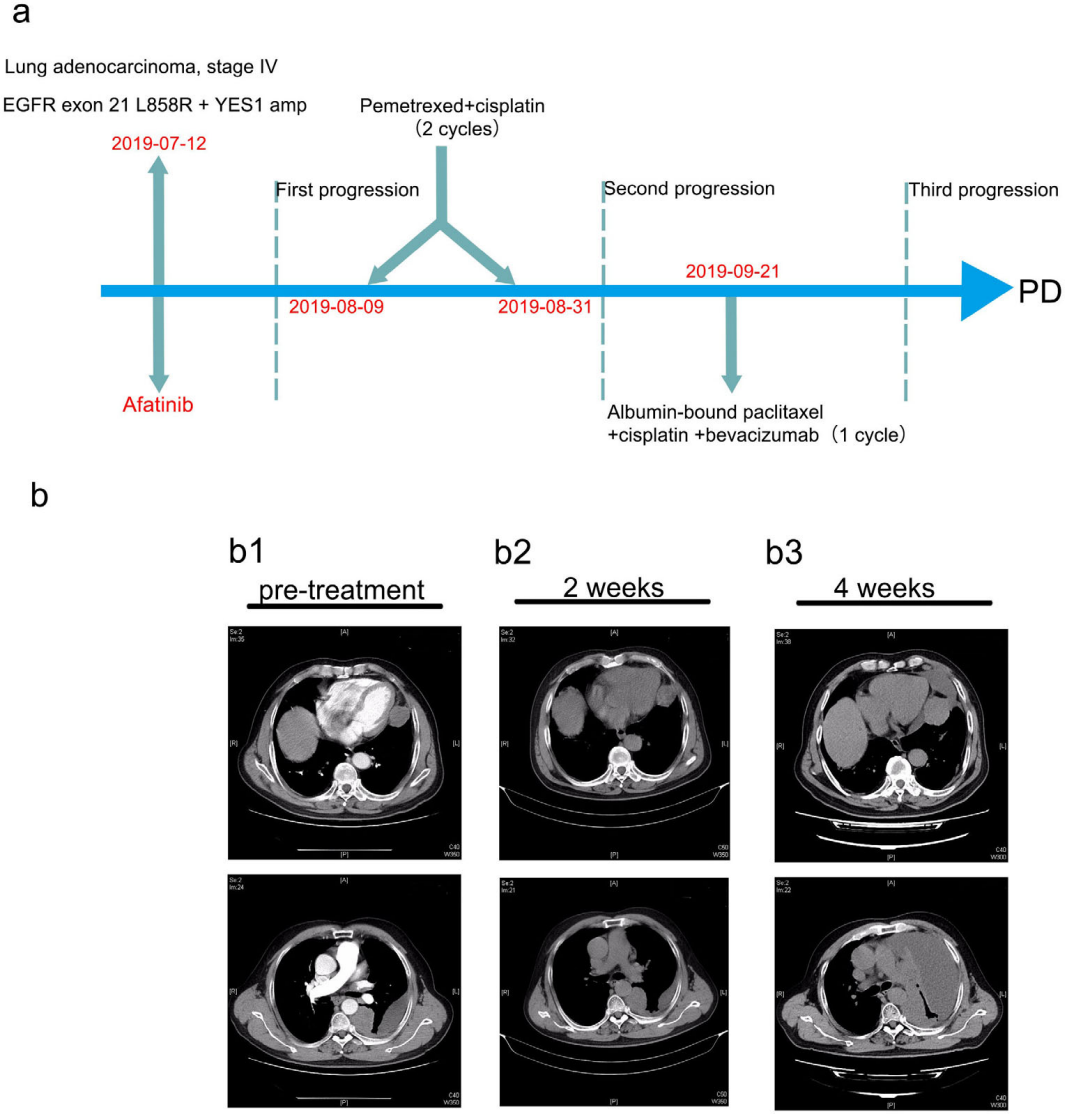
(**a**) A brief introduction to the treatment history. (**b**) Chest computed tomography (CT) scans. (**b1**) Preprogression. (**b2**) At two weeks; (**b3**) At four weeks.

A 68‐year‐old never‐smoking male who complained of left chest pain without apparent cause visited our hospital on 23 June 2019. He was diagnosed with stage IV lung adenocarcinoma with intrapulmonary, osseous and mediastinal lymph node metastasis and pleural effusion as shown in Fig 1a. Next‐generation sequencing (NGS) examination was performed to evaluate the genomic alterations of this patient before treatment and the results of NGS examination are provided in Table 1. It revealed that the patient harbored the missense mutation in exon 21 p.L858R of *EGFR*, *YES1* amplification,* CCND1* amplification,* FGF19* amplification, *FGF3* amplification and *FGF4* amplification.

**Table 1:** Next‐generation sequencing (NGS) results

Gene name	Alteration	Abundance	Targeted drug (Sensibility. evidence level)
*EGFR*	exon 21 p.L858R missense mutation	25.23%	Gefitinib (Sensibility. A) Erlotinib (Sensibility. A) Afatinib (Sensibility. A) Dacomitinib (Sensibility. A) Icotinib (Sensibility. A) Osimertinib (Sensibility. A)
*YES1*	Amplification	CN: 3.0	Dasatinib (Sensibility. C) Gefitinib (resistance. D) Erlotinib (resistance. D) Afatinib (resistance. D) Crizotinib (resistance. D)
*CCND1*	Amplification	CN: 3.6	Pembrolizumab (resistance. D) Nivolumab (resistance. D)
*FGF19*	Amplification	CN: 4.0	Pembrolizumab (resistance. D) Nivolumab (resistance. D)
*FGF3*	Amplification	CN: 3.9	Pembrolizumab (resistance. D) Nivolumab (resistance. D)
*FGF4*	Amplification	CN: 3.8	Pembrolizumab (resistance. D) Nivolumab (resistance. D)

NGS assay prior to EGFR‐TKI therapy. The genes without certain clinical significance were omitted. In this case, NGS assay was performed by Burning Rock Biotech based on Illumina sequencing platform.

The patient received first‐line afatinib therapy on 12 July 2019, but chest computed tomography (CT) on day 14 of treatment showed a slight enlargement of the left primary tumor. However, after four weeks of afatinib treatment, the tumor had enlarged significantly and the pleural effusion increased rapidly, resulting in a classification of progressive disease (PD) as shown in Fig 1b, and as evaluated according to Response Evaluation Criteria in Solid Tumors 1.1 (RECIST 1.1). After two cycles of second‐line chemotherapy including pemetrexed plus cisplatin, the patient experienced further PD. The patient was administered third‐line therapy of the regimen which consisted of albumin‐bound paclitaxel, cisplatin and bevacizumab. Unfortunately, the patient's disease was evaluated as PD after only one cycle treatment of this regimen.

## Discussion

Approximately 37.5%–51.4% of Chinese patients with advanced NSCLC have been found to have somatic activating mutations in *EGFR*.^1^, ^2^, ^3^ Patients with NSCLC who have somatic mutations of the *EGFR* gene have been found to benefit from EGFR‐TKIs including gefitinib, erlotinib, afatinib, dacomitinib, osimertinib and so on. In previous studies, targeted therapies have led to longer progression‐free survival (PFS) compared to treatment with platinum‐based chemotherapy.^4^, ^5^, ^6^ However, about 20% of NSCLC patients have less or no benefit from EGFR‐TKIs because of various primary resistance mechanisms. In this case, the *EGFR*‐positive NSCLC patient harboring *YES1* amplification demonstrated a primary resistance to afatinib.

Several mechanisms have been reported to be associated with decreased sensitivity to EGFR‐TKIs in *EGFR* mutation patients, including T790M mutation,^7^ exon 20 insertion^8^，*MET* amplification,^9^ overexpression of hepatocyte growth factor,^10^ overexpression of *NOTCH3*,^11^ anaplastic lymphoma kinase (*ALK*) positive,^12^ loss of* PTEN*,^13^ activation of* IGFR* signaling,^14^ *NF‐κB* pathways,^15^ and *STAT3* signaling.^16^ In this case, the genetic test report showed the patient harbored the missense mutation in exon 21 p.L858R of *EGFR* (25.23%), *YES1* amplification (CN:3.0),* CCND1* amplification (CN:3.6), *FGF19* amplification (CN:4.0), *FGF3* amplification (CN:3.9) and *FGF4* amplification (CN:3.8). *FGF3*, *FGF4*, *FGF19* and *CCND1* were co‐localized on the same chromosomal region (11q13). Previous studies have shown no significant difference in overall survival or ‐median PFS for first‐line therapy in patients with *FGF/FGFR*‐aberrant or wild‐type tumors, and that EGFR inhibitors could reduce CCND1 expression via eIF2α phosphorylation.^17^, ^18^ In the case reported here, we therefore conclude that *YES1* amplification, rather than *FGF*/*CCND1* might be the critical cause of primary resistance to afatinib.

*YES1* is a member of the SRC family kinases (SFKs) which regulate the proliferation, survival, angiogenesis, invasion and migration of cancer cells.^19^ Research has shown that YES1 plays a role in nuclear translocation of EGFR^20^ and acquired resistance to EGFR inhibitors in EGFR mutation positive NSCLC.^21^ However, the relationship between *YES1* amplification and primary resistance of EGFR‐TKIs remains unclear.

*YES1* amplification has been found in 15% of lung adenocarcinoma and 25% of lung squamous cell carcinoma patients, and *YES1* expression was reported to be related to a shorter OS in NSCLC patients.^22^ Garmendia *et al*.^23^ demonstrated that *YES1* amplification induces tumor growth as an oncogenic driver alteration in NSCLC. High YES1 protein expression was an independent predictor for poor prognosis in patients with NSCLC, and they indicated that SFKs may serve as potential therapeutic targets by the examination of *YES1* genetic alteration in NSCLC.

On 12 January 2018, the Food and Drug Administration (FDA) granted approval to afatinib for a broadened indication in the first‐line treatment of patients with metastatic NSCLC whose tumors had nonresistant *EGFR* mutations as detected by an FDA‐approved test. Therefore, in the future, there will be a greater choice of EGFR‐TKIs for clinicians to treat patients with *EGFR* mutation‐positive tumors. However, our case suggested that afatinib, including other first line EGFR‐TKIs, should be given carefully to patients harboring *EGFR* mutations combined with *YES1* amplification.

Few cases of primary resistance to afatinib for patients harboring *EGFR* exon 21 L858R missense mutation have been reported and here we present the first case with concurrent alterations of *EGFR* exon 21 L858R missense mutation and *YES1* amplification. Garmendia *et al*.^23^ demonstrated that *YES1* amplification presented a high sensitivity to dasatinib, an SFK inhibitor. Another study revealed that the disruption of the SFK pathway may remain a viable method of overcoming TKI resistance.^24^ Dasatinib combined with EGFR‐TKIs treatment may therefore benefit patients with concurrent alteration of *YES1* amplification and *EGFR* sensitive mutation but this should be verified in future studies.

In conclusion, we suggest that *YES1* mutation status should be assessed before EGFR‐TKIs treatment in patients with NSCLC harboring *EGFR* sensitive mutation in clinical practice, and we believe that clinical research and trials of dasatinib combined with EGFR‐TKIs in the use of patients with concurrent alteration of *YES1* amplification and *EGFR* mutation‐positive are warranted in the future.

## Disclosure

The authors report no conflicts of interest.
